# Supplementary material for: Reversible excision of the wzy locus in Salmonella Typhimurium may aid recovery following phage predation
Source: PLoS Genet. 2025 May 2;21(5):e1011688. doi: 10.1371/journal.pgen.1011688 (PMC12074656; doi:10.1371/journal.pgen.1011688)
Supplement: S1 File — Commands used for running Snippy, RAxML, Pyseer, and R scripts used for ancestral state reconstruction with phytools and simmap, and pastML. (DOCX) [file pgen.1011688.s006.docx]

**Snippy command (read alignment and snp calling)**

**snippy --R1**<in read1**> --R2** <in read2> **--ref** <reference.fasta>

**RAxML command**

**raxmlHPC -s** <input aln> **-n** <output suffix> **-m** GTRGAMMA **-T** 4 **-f** a **-x** 123 **-N** 1000 **-p** 456

With -f a : rapid Bootstrap analysis and search for best scoring ML tree in one program run

-p Specify a random number seed for the parsimony inferences. This allows

to reproduce your results and will help me debug the program.

-x being a random seed for resampling

**Pyseer command**

**#get kmers**

**pyseer --lmm** **--phenotypes** <phenotype table> **--kmers** <fsm_kmers> **--similarity** <mash table> **--output-patterns** <pattern out> **--cpu 4 >** <kmer out>

--lmm linear mixed model

**#Filtering your Output**

**filter_seer -k** <kmer out> **--pos_beta** **--maf** 0.05 **--sort** pval **>** <filtered_kmers.txt>

**#Filtered SEER output to fastq**

**hits_to_fastq.pl -k** <filtered_kmers.txt> **-b** 10e-8 **>** <output.fastq>

**R scripts :**

**##script 1**

**#this script is for ancestral reconstruction using phytools & simmap**

library(phytools)

library(ggplot2)

**#read finalised dt8 tree**

dt8 <- read.tree("DT8_tree_drop_tips.newick")

**#plot tree**

plotTree(dt8, ftype="i", fsize = 0.4, type = "fan")

**#read phage type data**

pt <- read.csv("Strain_phagetype.csv", row.names=1)

**#make into matrix**

ptd <- as.matrix(pt)[,1]

**#or an MCMC tree displaying the ancestral histories**

mtree<-make.simmap(dt8,ptd,model="ARD")

mtree

plot(mtree,cols,fsize=0.4,ftype="i", type = "fan")

add.simmap.legend(colors=cols,prompt=FALSE,x=0.9*par()$usr[1],

y=-max(nodeHeights(dt8)),fsize=0.5)

**#make pdf of MCMC tree**

pdf("dt8_MCMC_anc_state_bl_gold.pdf")

plot(mtree,cols,fsize=0.4,type = "fan")

add.simmap.legend(colors=cols,prompt=FALSE,x=0.9*par()$usr[1],

y=-max(nodeHeights(dt8)),fsize=0.5)

dev.off()

**#this doesnt mean much on its own, need to look at distribution among many simulated trees**

**#making 1000**

mtrees<-make.simmap(dt8,ptd,model="ARD",nsim=1000, Q = "mcmc")

pd<-summary(mtrees,plot=FALSE)

pd

**#also making a density map of the 1000 simulated stochastic trees**

obj2<-densityMap(mtrees,lwd=4, outline = FALSE, fsize = 0.4, type = "fan")

#pdf of this

pdf("density_map_dt8_tree_post_probs_no_outline.pdf")

densityMap(mtrees,lwd=5, outline = FALSE, fsize = 0.4, type = "fan")

dev.off()

**#tree above used for figure (edited in illustrator to remove tip, rotate to fit pastML layout,**

**#and make branch thinner)**

**##script 2**

**##this script is for ancestral reconstruction using phytools**

library(phytools)

**#read finalised dt8 tree**

dt8 <- read.tree("DT8_tree_drop_tips.newick")

#read phage type data

pt <- read.csv("Strain_phagetype.csv", row.names = 1)

**#arguments for loop**

startpermut=1 #what number to start from

endpermut=20 #number of last permutation

nsimulation=100 #number simulation for simmap step. higher number --> takes longer to run

for (i in startpermut:endpermut){

print(paste("processing permutation",i))

**#permute data (re-assigne phage type randomly)**

permut_phagetype<-pt

permut_phagetype$Phage.Type<-pt[sample(nrow(pt)),]

#build ancestral state using ARD model and permuted data

fitARD_permut<-ace(as.matrix(permut_phagetype),

dt8,type="discrete",

CI = TRUE, model = "ARD")

print(paste("log likelyhood",fitARD_permut$loglik))

print(paste("running probability tree for permutation",i,"with",nsimulation,"simulations, may take a while"))

permut_tree<-make.simmap(dt8,as.matrix(permut_phagetype)[,1],nsim=nsimulation, q = "mcmc")

sumtree<-summary(permut_tree,plot=FALSE)

write.csv(as.data.frame(sumtree$ace), paste("permutation_",i,"_nodes.csv",sep=''))

print("done, plotting density map")

pdf(paste("density_permutation_",i,".pdf",sep=''))

densityMap(permut_tree,lwd=4, outline = FALSE, fsize = 0.3, type = "fan" )

dev.off()

print(paste("permutation",i,"done"))

}

**## script 3**

**#this script is for plotting permutations of DT8 ancestral reconstruction data**

**#requires to create permutation first (see create_permutation.R)**

**#read data file**

d <- read.csv("permutation_all_nodes.csv")

**#load packages**

library(ggplot2)

library(reshape2)

**#melt data frame into plottable format**

dm <- melt(d)

**#plot as transparent boxplot with dots**

perm_plot <- ggplot(dm, aes(x=dm$variable, y = dm$value)) +

geom_boxplot(size = 0.5, fill = NA, color = "black")+

# geom_jitter(width = 0.14, size = 0.5, color="dark blue") +

xlab("permutation") +

ylab("Probability of node being DT30")+

theme_minimal()+

theme(axis.text.x = element_text(angle = 90))

perm_plot

ggsave("permutation_tests_DT30_nodes.pdf", height = 7, width = 7, dpi = 1000, useDingbats = F)

**#undertake non-parametric comparison tests**

wilcox.test(d$Data, d$permutation.1)

**# significantly different**

wilcox.test(d$Data, d$permutation.2)

**# significantly different**

wilcox.test(d$Data, d$permutation.3)

**# significantly different**

wilcox.test(d$Data, d$permutation.4)

**# significantly different**

wilcox.test(d$Data, d$permutation.5)

**# significantly different**

wilcox.test(d$Data, d$permutation.6)

**# significantly different**

wilcox.test(d$Data, d$permutation.7)

**# 0.056**

wilcox.test(d$Data, d$permutation.8)

**# significantly different**

wilcox.test(d$Data, d$permutation.9)

**# significantly different**

wilcox.test(d$Data, d$permutation.10)

**# 0.07144**

wilcox.test(d$Data, d$permutation.11)

**# signifncantly different**

wilcox.test(d$Data, d$permutation.12)

**# signifncantly different**

wilcox.test(d$Data, d$permutation.13)

**# significantly different**

wilcox.test(d$Data, d$permutation.14)

**# significantly different**

wilcox.test(d$Data, d$permutation.15)

**# significantly different**

wilcox.test(d$Data, d$permutation.16)

**# significantly different**

wilcox.test(d$Data, d$permutation.17)

**# signifncantly different**

wilcox.test(d$Data, d$permutation.18)

**# signifncantly different**

wilcox.test(d$Data, d$permutation.19)

**# signifncantly different**

wilcox.test(d$Data, d$permutation.20)

**#signifcantly different**

**##script 4**

**#plot DT30 state probability from pastML vs ace prediction**

**#load DT30 node probbilities from SIMMAP and MPPA estiamte**

DT30 <- read.csv("DT30_node_probabilities.csv", row.names=1)

**#generate linear model**

lm(DT30$DT30_MPPA ~DT30$DT30_SIMMAP)

**#intercept = 0.1106 + 0.8377**

**#plot using ggplot**

library(ggplot2)

plot <- ggplot(DT30, aes(x=DT30$DT30_SIMMAP, y=DT30$DT30_MPPA))+

geom_point(colour = "dark blue")+

theme_minimal()+

xlab("DT30 state probabilities from MPPA")+

ylab("DT30 state probabilities from stochastic mapping")

plot

**#add r-squared and line**

require(stats)

abline(fit <- lm(DT30$DT30_MPPA ~ DT30$DT30_SIMMAP), lty="dashed", col="red",lwd=2)

fit

coef = coefficients(fit)

eq <- paste0("y = ", round(coef[2],digits = 2), "*x + ", round(coef[1],digits = 2)," R2=0.675")

plot + geom_abline(intercept = 0.1106, slope= 0.8377, colour = "red", linetype = "dashed", size = 1) + ggtitle(eq)

**#add R squared value**

c<- cor(DT30$DT30_SIMMAP,DT30$DT30_MPPA)

rsq <- function (x, y) cor(x, y) ^ 2

rsq(DT30$DT30_SIMMAP, DT30$DT30_MPPA)

pdf("linear_model_DT30_node_probabilities.pdf")

plot <- ggplot(DT30, aes(x=DT30$DT30_SIMMAP, y=DT30$DT30_MPPA))+

geom_point(colour = "dark blue")+

theme_minimal()+

xlab("DT30 state probabilities from MPPA")+

ylab("DT30 state probabilities from stochastic mapping")+

geom_abline(intercept = 0.1106, slope= 0.8377, colour = "red", linetype = "dashed", size = 1) + ggtitle(eq)

plot

dev.off()

**##Script 5**

**#this script is to test and compare models using ace and phytools**

library(phytools)

**#read finalised dt8 tree**

dt8 <- read.tree("DT8_tree_drop_tips.newick")

**#read phage type data**

pt <- read.csv("Strain_phagetype2.csv", row.names=1)

**#make into matrix**

ptd <- as.matrix(pt)[,1]

**#lik.anc gives us marginal ancestral states, or "empirical Bayesian posterior probabilities" this is using an all-rates-different substitution matrix, more like felsenstein for nuceic acid substitution.**

fitARD<-ace(ptd,dt8,type="discrete", CI = TRUE, model = "ARD")

fitARD$loglik

**#we can try this with an equal rates model (e.g jukes cantor if talikng about nucleotide substitution)**

fitER<-ace(ptd,dt8,type="discrete", CI = TRUE, model = "ER")

fitER$loglik

**#p value for LRT with ML trees**

LRT_ML_p_val <- (1 - pchisq(2*abs((fitER$loglik) - (fitARD$loglik)), 1))

LRT_ML_p_val

**#using phytool, either mcmc or ARD**

mtrees100mc<-make.simmap(dt8,ptd,nsim=100, Q = "mcmc")

mtrees100ard<-make.simmap(dt8,ptd,nsim=100, Q = "ARD")

**#make a summary of these trees**

pd<-summary(equaltrees,plot=FALSE)

pd

**#extract the log likelihoods to use in LRT test**

equal_loglik <- data.frame()

equal_loglik[1,] <- equaltrees[[1]]$logL

for(i in 1:100){equal_loglik[,i] <- equaltrees[[i]]$logL}

equal_loglik <- as.numeric(equal_loglik)

eqav <- mean(equal_loglik)

**#and a summary of the MCMC map**

pd2 <- summary(mtrees100mc,plot = FALSE)

pd2

**#extract the log likelihoods to use in LRT test**

mcmc_loglik <- data.frame()

mcmc_loglik[1,] <- mtrees100mc[[1]]$logL

for(i in 1:100){mcmc_loglik[,i] <- mtrees100mc[[i]]$logL}

mcmc_loglik <- as.numeric(mcmc_loglik)

mcav <- mean(mcmc_loglik)

LRT_stochastic_maps_p_value <- (1-pchisq(2*abs(mcav - eqav), 1))

LRT_stochastic_maps_p_value

**#1.88x10^-15**
